# Ankylosing spondylitis, chronic fatigue and depression improved after stromal vascular fraction treatment for osteoarthritis: a case report

**DOI:** 10.1186/s13256-018-1776-y

**Published:** 2018-08-29

**Authors:** Bora Bright, Ralph Bright, Pelin Bright, Amita Limaye

**Affiliations:** Macquarie Stem Cells, 21b Bathurst Street, Liverpool, NSW 2170 Australia

**Keywords:** Osteoarthritis, Ankylosing spondylitis, Depression, Stromal vascular fraction, Adipose derived, Mesenchymal stem cells

## Abstract

**Background:**

Osteoarthritis is a prevalent chronic disease that impacts quality of life and imposes a heavy economic burden. Despite this there is no confirmed treatment that could prevent progressive destruction of osteoarthritic joints. Mesenchymal stem cells with their regenerative and immunosuppressive properties have emerged as a potential therapy.

**Case presentation:**

This case study describes the impact of autologous adipose-derived stromal vascular fraction treatment on a 27-year-old Australian woman with osteoarthritis and multiple comorbidities of ankylosing spondylitis, chronic pain syndrome, and post-traumatic stress disorder as assessed by magnetic resonance imaging, Western Ontario and McMaster Universities Osteoarthritis Index, and Hip Disability and Osteoarthritis Outcome Score. Following standardized stromal vascular fraction treatment protocols for osteoarthritis of her hips and knee, the functional status of her hips was measured by Hip Disability and Osteoarthritis Outcome Score at 3 months, 6 months, and 3 years.

**Conclusions:**

Our patient showed dramatic improvements to her quality of life and symptoms of osteoarthritis were reduced. Interestingly, along with improvements in her knee and hips her other comorbidities such as ankylosing spondylitis, depression, anxiety, and fatigue exhibited marked improvement. She ceased the use of a wheelchair and walking support and, with increased mobility, had gained independence. These findings are suggestive of the therapeutic effects of stromal vascular fraction.

## Background

Osteoarthritis (OA) is characterized by degeneration of articular cartilage, sclerosis of the subchondral bone, and marginal osteophyte formation. OA is associated with chronic pain, stiffness, decreased range of motion and joint deformity, muscle wasting, and tendonitis. The diagnosis of OA relies on clinical symptoms, physical findings, and radiographic findings. Discordance is often found between radiological findings and symptomatic findings of OA [1]. The etiology of OA appears to be multifactorial with hereditary, metabolic, hormonal, developmental, and mechanical components [2]. Apart from mechanical and genetic factors that contribute to development of OA, age has been a primary risk factor [3]. Age-associated changes in cartilage extracellular matrix (ECM), increase in inflammatory cytokine milieu locally, and increased free radical species have been implicated in the loss of ability of cartilage to adapt to mechanical stress or load [4]. Interestingly, depletion of the mesenchymal stem cells (MSCs) in local stromal population has been indicated to be associated with OA [5, 6]. Whether it is a cause or an effect of OA remains to be elucidated. Current treatments include lifestyle modifications and diet together with pain relief using paracetamol or non-steroidal anti-inflammatory drugs (NSAIDs) in early OA. However, treatment of the advanced disease relies on total joint replacement which is found to be associated with complications. The lifespan of the prosthesis is limited; therefore, surgery is delayed until symptoms limit a patient’s lifestyle and is avoided in younger patients. Since most of the abovementioned modalities only provide symptomatic relief, the regenerative potential of stem cells to repair injured and damaged tissue is a promising new strategy in the field of orthopedics. Of these options, patient-derived (autologous), minimally manipulated, MSCs, for the treatment of chronic diseases such as OA is being investigated to achieve clinical significance [7].

Here we describe a case report on the healing effect of adipose-derived stromal vascular fraction (SVF) in a woman with peripheral spondyloarthritis, grade 2 sacroiliitis, enthesitis, and ankylosing spondylitis (AS) that were confirmed by X-ray, magnetic resonance imaging (MRI), computed tomography (CT) scan, and ultrasound scan. AS is a form of arthritis that primarily impacts the spine, causing inflammation, leading to chronic pain. AS symptoms often include disturbances of sleep, fatigue, depression, and anxiety [8]; therefore, they have a profound impact on the patient’s quality of life. AS is predominantly detected in patients between the ages of 20 and 40 years and can be triggered by multiple factors that are similar to those for OA. Conditions associated with inflammation such as OA and AS are controlled by NSAIDs and common analgesic medication [9].

MSCs can be found throughout the body. Adipose tissue is an excellent source of stem cells having 10–100 times more MSCs than bone marrow [10]. SVF can be easily obtained from loose connective tissue that is associated with adipose tissue by a process of liposuction under local anesthesia. SVF is a collection of a heterogeneous population of: MSCs; hematopoietic stem cells (HSCs); regulatory T cells (T_reg_); pericyte-endothelial cells (ECs); mast cells; a complex microvascular structure of fibroblasts, white blood cells (WBC), dendritic cells (DCs), and intra-adventitial smooth muscle-like cells; and ECM. The immunomodulatory, anti-inflammatory, and regenerative properties of SVF are not attributed to a single type of population residing within, although they are effects of all the types of cells constituting the SVF. The use of SVF as a medical treatment is increasing due to the abundance of its cellular properties, ease of collection, immunomodulatory properties, and safety [11].

The aim of this treatment was to reduce pain associated with OA and improve our patient’s quality of life without the need for NSAIDs or analgesics. Our patient was expected to show significant improvements in her OA, along with some minor improvements to general inflammation present within her body. Interestingly, short-term follow-up presented improvements in her OA as well as comorbidities of AS, depression, anxiety, and fatigue. A 3-year follow-up (in June 2017) including multiple injections of SVF indicated significant changes in her quality of life. All conditions maintained their improvements at the follow-up intervals.

## Case presentation

Our patient was a 27-year-old Australian woman with grade IV OA confirmed by X-ray images of her pelvis; ultrasound scans showed right knee joint effusion, enthesitis, and synovitis; a CT scan of her spine indicated annulus bulges at L3/4 and L4/5, and bilateral grade 2 sacroiliitis changes; a background of AS (human leukocyte antigen-B27 negative) confirmed by MRI imaging; chronic pain syndrome with pain amplification; and post-traumatic stress disorder. Her body mass index (BMI) was 39.4 kg/m^2^. She did not have any: infection with hepatitis B, hepatitis C, or human immunodeficiency virus (HIV); malignancy; previous history of allergic reaction to any component of our therapeutic measure; active cardiac, respiratory, neurologic or endocrine disease necessitating receipt of medication. She was not pregnant or in lactating condition. A written and informed consent was obtained from our patient. Arthritic symptoms were measured using Western Ontario and McMaster Universities Osteoarthritis Index (WOMAC) and Hip Disability and Osteoarthritis Outcome Score (HOOS) by scoring for pain intensity, walking ability (distance), joint stiffness, physical function, sports and recreation, and quality of life. Changes to her AS symptoms were measured using the Ankylosing Spondylitis Quality of Life (ASQoL) questionnaire. For liposuction and stem cell treatment, she was admitted to Macquarie Stem Cells. Under light sedation and using aseptic technique, 450 ml of fat was harvested from her abdomen. Cell isolation was performed in PC II safety cabinet. Cells were isolated using collagenase digestion using Liberase GMP grade (enzyme blend).

**Fig. 1:**
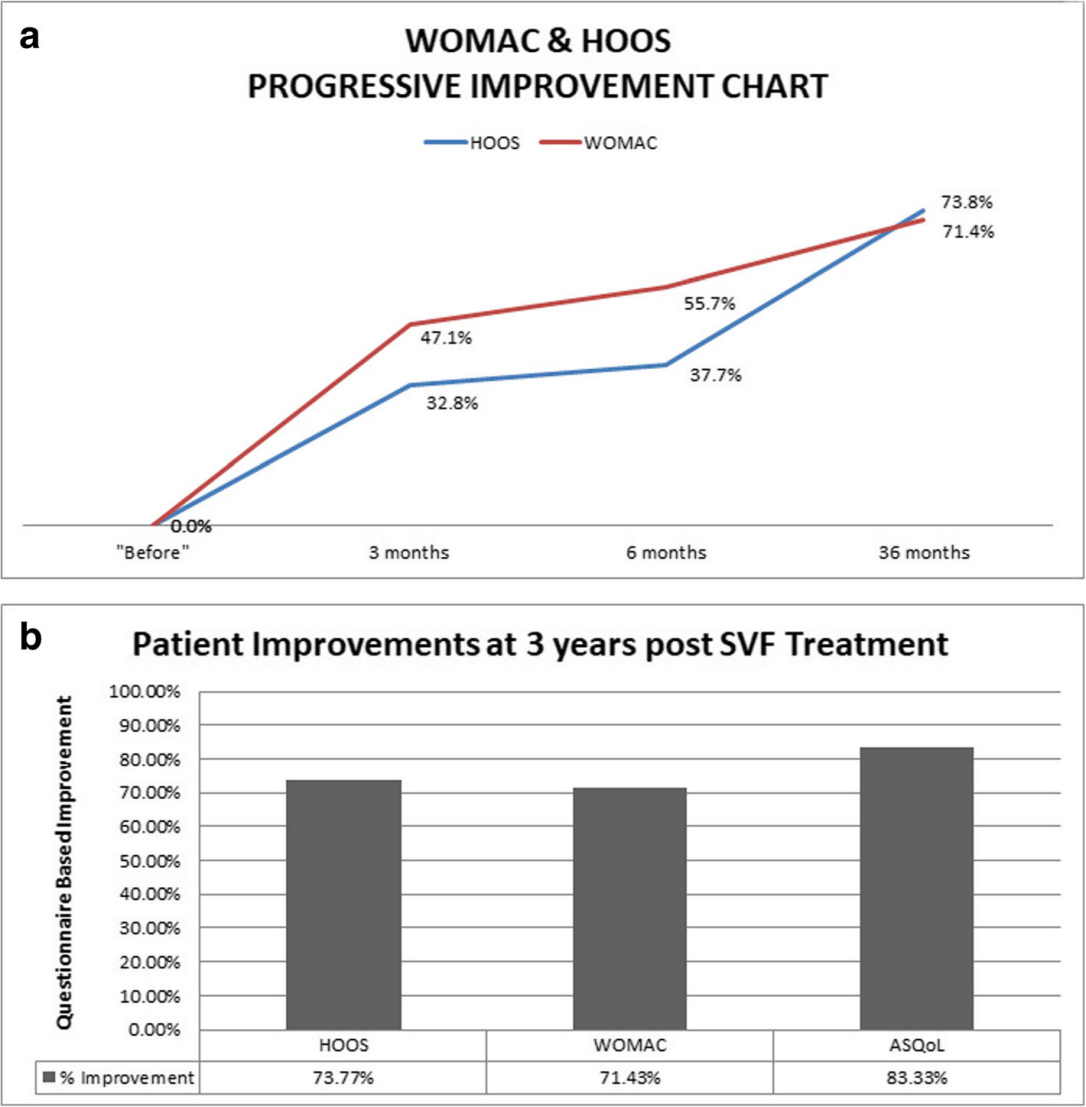
Western Ontario and McMaster Universities Osteoarthritis Index and Hip Disability and Osteoarthritis Outcome Score progressive improvement chart. **a** Percentage improvement of Western Ontario and McMaster Universities Osteoarthritis Index (*red*) and Hip Disability and Osteoarthritis Outcome Score (*blue*) on *Y*-axis over 36 months (on *X*-axis). The graph highlights the measurements taken at 0 (baseline or pre-treatment score), 3 months, 6 months and at 36 months after first stromal vascular fraction infusion. **b** Patient improvement based on subjective questionnaires after stromal vascular fraction treatment. Improvement of patient in terms of Western Ontario and McMaster Universities Osteoarthritis Index and Hip Disability and Osteoarthritis Outcome Score that measure osteoarthritis and Ankylosing Spondylitis Quality of Life questionnaire that measures quality of life of a patient with ankylosing spondylitis over baseline. The percentage improvement over baseline (on *Y*-axis) and questionnaires used in the study (on *X-*axis). *ASQoL* Ankylosing Spondylitis Quality of Life, *HOOS* Hip Disability and Osteoarthritis Outcome Score, *SVF* stromal vascular fraction, *WOMAC* Western Ontario and McMaster Universities Osteoarthritis Index

Our patient’s preoperative HOOS score (baseline score) for both hips was 122 (range 0–168), WOMAC for her right knee was 70 (range 0–90), and the baseline ASQoL questionnaire was 18 (range 0–18). We obtained 2.058 billion nucleated cells with a viability of 89.10% using Muse® Cell Analyzer. A total of 738 million cells were injected on the day: 100 million cells injected into each hip and right knee intra-articular under ultrasound guidance, and 438 million cells were administered as an intravenous infusion. The remaining 1.320 billion cells were cryogenically frozen into four separate vials of 330 million cells following the protocols of Thirumala *et al.* [12]. Follow-up intravenous infusions of 330 million cells were provided at 3 months, 12 months, and 36 months. Our patient’s follow-up intervals were performed at 1 day, 3 months, 6 months, 12 months, 24 months, and 36 months respectively. Neither local nor systemic adverse events were observed during the follow-up and she was satisfied with the therapy after 3 months with an increasing trend over the period. At 3-month post-treatment, she exhibited increased mobility. Her HOOS and WOMAC scores decreased to 82 and 37, respectively from her baseline scores. She also noted that pain in her spine, hips, and right knee associated with OA and AS had decreased. Interestingly, in addition to her decreased pain and increased mobility, she was feeling more energetic. Within 6 months after the first SVF infusion, her HOOS and WOMAC questionnaire scores had decreased to 79 and 31, respectively. She showed dramatic improvements over 2 years after her first SVF infusion and presented with decreased dependency on a wheelchair or walking stick (HOOS and WOMAC scores not available). Her dependency on pain relief and anti-depressant medications was found to be decreased as is evident from Table 1. At the 36-month follow-up, she presented significant improvements overall. She remained free from NSAIDs and her pain levels were minimal. Follow-up HOOS and WOMAC scores had decreased to 32 and 20, respectively. Her pre-treatment to post-treatment ASQoL score had decreased to 3 signifying increased quality of life. She still presents some symptoms of depression; however, her anxiety appears to have resolved almost completely. Her progressive improvement is observed over 3 years with WOMAC, HOOS, and ASQoL (Fig. 1a, b).

**Table 1:** Medical history timeline

Timeline	2006	2008	2011	2012	2014	2014	2015	2016	2017
Age	19 years	21 years		25 years	27 years	27 years	28 years	29 years	30 years
Disease	AS (HLA-B27 negative)	MVA		OA	OA grade IV	OA grade IV			
Symptoms	Spinal pain involving shoulders and interscapular region and lower lumbar spine	Severe fracture of right knee		Effusion, reduced range of movement, low grade enthesitis, bilateral grade 2 sacroiliitis, peripheral arthritis	Effusion, reduced range of movement, low grade enthesitis, bilateral grade 2 sacroiliitis, peripheral arthritis	Effusion, reduced range of movement, low grade enthesitis, bilateral grade 2 sacroiliitis, peripheral arthritis, OA of hips and right knee			
Diagnosis	MRI			CT scan and ultrasound scan		X-ray			
Medication as per medical history	Data not available	Data not available	Allegron (nortriptyline; 25 mg X 2)	Xenacort A (Triamcinolone acetonide)40	Allegron (nortriptyline; 25 mg X 2)	Allegron (nortriptyline; 25 mg X 1)	Allegron (nortriptyline)		
			Mirtazon (mirtazapine; 30 mg)	Ketoprofen (200 mg)	Mirtazon (mirtazapine; 30 mg)	Mirtazon (mirtazapine; 30 mg)	Mirtazon (mirtazapine)		
			Cymbalta (duloxetine; 60 mg X 5)	Cymbalta (duloxetine; 60 mg X 3)	Cymbalta (duloxetine; 60 mg X 3)	Cymbalta (duloxetine; 60 mg X 3)	Cymbalta (duloxetine)	Valdoxan (agomelatine; 25 mg X 3)	
			Endone (oxycodone; 5 mg X 3)	Diazepam (5 mg X 3)	Endone (oxycodone; 5 mg X 3)	Endone (oxycodone; 5 mg X 3)	Endone (oxycodone)	Tramadol (50 mg X 2, 150 mg X 1)	
			Lyrica (pregabalin; 150 mg X2)	Endone (oxycodone; 5 mg X 4)	Lyrica (pregabalin; 150 mg X 5)	Lyrica (pregabalin; 150 mg X 3)	Lyrica (pregabalin)	Lyrica (pregabalin; 300 mg X 2)	
			Nexium (esomeprazole; 40 mg)	Lyrica (pregabalin; 150 mg X 2)	Nexium (esomeprazole; 40 mg)	Nexium (esomeprazole; 40 mg)	Nexium (esomeprazole)		
			Orudis SR (ketoprofen; 200 mg X 3)	Nexium (esomeprazole; 40 mg)	Orudis SR (ketoprofen; 200 mg X 3)	Orudis SR (ketoprofen; 200 mg)	Orudis (ketoprofen)		
			Oxycontin (oxycodone; 30 mg)		Oxycontin (oxycodone; 30 mg)	Oxycontin (oxycodone; 30 mg)	Oxycontin (oxycodone)		
			Panadol Osteo (paracetamol; 665 mg PRN)	Remeron (mirtazapine; 30 mg)	Panadol Osteo (paracetamol; 665 mg PRN)	Panadol Osteo (paracetamol; 665 mg PRN)	Panadol Osteo (paracetamol)	Somac (pantoprazole; 20 mg X 1)	
			Seroquel (quetiapine; 25 mg X 3)	Panadol Osteo (paracetamol; 665 mg × 3) PRN)	Seroquel (quetiapine; 25 mg X 3)	Seroquel (quetiapine; 25 mg X 3)	Seroquel (quetiapine; 25 mg X 3)	Seroquel (quetiapine; 25 mg X 3)	
				Seroquel (quetiapine; 25 mg)	Ostelin (ergocalciferol; 25 mcg)	Ostelin (ergocalciferol; 25 mcg)	Ostelin (ergocalciferol)	Ostelin (ergocalciferol)	
					Pantoprazole	Pantoprazole	Pantoprazole		
					Physiotens(moxonidine	Physiotens (moxonidine)	Physiotens (moxonidine		
					Valium (diazepam; 5 mg PRN)	Valium (diazepam; 5 mg PRN)	Valium (diazepam)		
					Methadone (10 mg X 6)	Methadone (10 mg X 6)	Methadone	Methadone (10 mg X 5)	
					Uremide (furosemide)	Uremide (furosemide)	Uremide (furosemide)		
					Tresos B multivitamin	Tresos B multivitamin	Tresos B multivitamin	Tresos B multivitamin	
					BioMagnesium high dose	BioMagnesium high dose	BioMagnesium high dose	BioMagnesium high dose	
					Humira (adalimumab) injection	Humira (adalimumab) injection	Humira (adalimumab) injection		
						Oroxine (levothyroxine)	Oroxine (levothyroxine)	Oroxine (levothyroxine)	
Walking aid				Wheelchair usage	Wheelchair usage	Wheelchair usage	Stick/Walker		
Intervention						SVF infusion July 2014 --> Oct 2014 --> Aug 2015 --> June 2017			

*AS* ankylosing spondylitis, *CT* computed tomography, *HLA* human leukocyte antigen, *MRI* magnetic resonance imaging, *MVA* motor vehicle accident, *OA* osteoarthritis, *PRN* as necessary, SVF stromal vascular fraction

## Discussion and conclusions

Consistent with our findings, positive therapeutic effects of SVF have been shown in treatment of OA of hip and knee [7]. In 2011, based on a case series of 339 patients treated with SVF, Centeno *et al.* reported that 69% of patients were candidates for knee replacement [13]. However, after treatment with MSCs only 6.9% took up the option for replacement. Out of all the patients, 60% reported > 50% pain relief and 40% reported > 75% pain relief at 11 months [13]. We report here that SVF infusion given intravenously as well as intra-articularly was not only safe but effective in alleviating the pain associated with grade IV OA of hips and knee. HOOS and WOMAC scores showed 73% improvement over baseline (Fig. 1b). HOOS and WOMAC questionnaires are both subjective measurements used to observe function following joint arthroscopy and arthroplasty procedures and are proven to be useful for the evaluation of patient-relevant outcomes [14]. Interestingly, her AS also showed improvements based on ASQoL: her ASQoL score decreased from 18 down to 3 after SVF treatment. We used the ASQoL to assess our patient’s AS; the ASQoL questionnaire is a feasible method of determining a patient’s quality of life [15].

This is a first study reporting improvement of AS, a comorbidity, along with OA. In 2014, Wang *et al.* conducted an AS allogeneic stem cell treatment 20-week follow-up (ASAS20) study, which involved treating patients with AS via intravenous infusion of allogeneic cells with a 20-week follow-up [16]. The findings confirmed improvements via both objective MRI evaluations as well as subjective questionnaires. The responders showed 77.4% improvement based on the questionnaire assessment, and the MRI reports confirmed a decrease in inflammation [16]. These results are similar to the findings of the study described here, except for the use of allogeneic bone marrow-derived stem cells. Allogeneic and autologous cells are similar in nature, however, previous publications have shown autologous cells can be better in performance and safety when compared to allogeneic cells [17, 18]. SVF contains a high number of adipose-derived stem cells (ASCs) that are reported to preferentially migrate toward injured, inflamed, or hypoxic tissues to promote regeneration [19]. Chemokines and cytokines play an important role in cell activation, survival, and differentiation as well as cell migration. Inflammation is a key biomarker in driving depression [20, 21]. SVF plays a key role in suppressing inflammation; it thereby aids in repair and regeneration. Medications such as Lyrica (pregabalin), Nexium (esomeprazole), and Orudis (ketoprofen) have been associated with minor side effects of depression. We need to consider that possible reasons for improved depression could be related to a decrease in medication use, along with the immunomodulatory effects of SVF. The hypothesis in improving a patient’s quality of life is not only due to improvements in arthritic changes, it can also be associated with suppressing the inflammatory biomarkers that are linked to depression. This original case report provides insight into the fact that SVF treatment has the potential to improve patients’ quality of life by improving joint function and mobility, and decrease pain in patients with OA as well as AS. Further study utilizing multiple patients is required to arrive at conclusions on the effectiveness of this treatment for AS.

## Abbreviations

AS: Ankylosing spondylitis
ASQoL: Ankylosing Spondylitis Quality of Life
CT: Computed tomography
ECM: Extracellular matrix
HOOS: Hip Disability and Osteoarthritis Outcome Score
MRI: Magnetic resonance imaging
MSCs: Mesenchymal stem cells
NSAIDs: Non-steroidal anti-inflammatory drugs
OA: Osteoarthritis
SVF: Stromal vascular fraction
WOMAC: Western Ontario and McMaster Universities Osteoarthritis Index
